# Belimumab to Aid Pre‐Transplant Immunological Risk‐Stratification by Uncovering Broader HLA‐Specific Memory B‐Cell Profiles

**DOI:** 10.1111/tan.70285

**Published:** 2025-06-16

**Authors:** Dennis A. J. van den Broek, Gonca E. Karahan, Soufian Meziyerh, Yvonne de Vaal, Kim H. Bakker, Geert W. Haasnoot, Joris I. Rotmans, Y. K. Onno Teng, Cees van Kooten, Sebastiaan Heidt, Dave L. Roelen, Aiko P. J. de Vries

**Affiliations:** ^1^ Division of Nephrology, Department of Internal Medicine Leiden University Medical Center (LUMC), Leiden University Leiden the Netherlands; ^2^ Leiden Transplant Center, Leiden University Medical Center, Leiden University Leiden the Netherlands; ^3^ Department of Immunology, HLA Laboratory Leiden University Medical Center (LUMC) Leiden the Netherlands; ^4^ Eurotransplant Reference Laboratory, Leiden University Medical Center Leiden the Netherlands; ^5^ Erasmus MC Transplant Institute, Department of Internal Medicine Erasmus Medical Centre Rotterdam the Netherlands

**Keywords:** antibody‐mediated rejection, BAFF inhibition, highly‐sensitised, HLA‐antibody, HLA‐specific memory B‐cell, renal transplantation, risk‐stratification

## Abstract

Highly sensitised kidney transplant candidates face substantial barriers to transplantation due to limited donor compatibility. Delisting unacceptable antigens with detectable HLA‐specific antibodies can improve transplant access. It is, however, challenging to determine which specificities are safe to delist. Assessment of circulating HLA‐specific memory B‐cells may support risk stratification. However, current methodologies are limited by the predominant localisation of memory B‐cells in secondary lymphoid organs and thus by potential false negativity. BAFF inhibitors, such as belimumab, mobilise memory B‐cells into the circulation, which could reduce false‐negative evaluations and improve pretransplant risk stratification. We administered off‐label treatment with 200 mg of subcutaneous belimumab weekly for 4 weeks to seven highly sensitised patients with limited allocation probability. The clinical aim was to safely increase allocation probability by selectively delisting unacceptable HLA‐specificities without detectable B‐cell memory. HLA‐specific memory B‐cell profiles were assessed before and after treatment, revealing significantly broader HLA‐specific memory B‐cell profiles after belimumab in addition to a significant increase of HLA‐antibody MFI in the eluate of stimulated memory B‐cells. This strategy may pave the way for a new paradigm in pretransplant immunological risk‐stratification, allowing improved assessment of HLA‐specific memory B‐cell profiles, which could potentially limit the risk of memory responses.

**Abbreviations**
AD‐BCR: antigen‐density corrected BCR
AM: acceptable mismatch
AMR: antibody‐mediated rejection
BAFF: B‐cell activating factor
BCM: background corrected mean MFI
BCR: The BCM divided by the raw MFI of the lowest ranked bead for a locus
CDC: complement‐dependent cytotoxicity assay
DSA: donor‐specific antibodies
ET: eurotransplant
GEE: generalised estimated equations
IRB: institutional review board
mBC: memory B‐cell
MFI: mean fluorescence intensity
PRA: panel‐reactive antibodies
SAB: single‐antigen bead assay
UA: unacceptable antigen
vPRA: virtual PRA

## 1 Introduction

Pretransplant sensitisation to HLA increases the risk of antibody‐mediated rejection (AMR) [1, 2]. This risk may be mitigated by designating HLA‐specificities as unacceptable antigens (UA) if circulating HLA‐antibodies have been detected. However, this approach reduces allocation probability, creating an unmet need for transplant access among highly sensitised patients with virtual panel‐reactive antibodies (vPRA) > 85% [3]. Priority programmes, such as the Eurotransplant Acceptable Mismatch (AM) Programme, aim to increase the likelihood of finding a compatible donor for highly sensitised patients, but not all candidates meet the acceptance criteria and allocation probability remains poor for patients with vPRA > 99% [4, 5, 6, 7]. Allocation probability may also be increased by “delisting” [2, 8, 9]. Delisting is the process of redefining UA to which circulating antibodies have been detected as acceptable. While this can increase donor availability for a given patient, it carries an increased risk of AMR if a donor organ expresses delisted antigens. Ideally, only specificities conveying the lowest humoral risk are delisted to ensure sufficient allocation probability for successful transplantation. Yet, in practice, this approach may fail to adequately reduce vPRA. Delisting of higher‐risk specificities may consequently need to be considered, often in conjunction with pretransplant desensitisation [10, 11], to improve the likelihood of finding a compatible donor.

In these cases, a stepwise approach seems logical [9]. HLA‐specificities classified as low‐risk should be delisted first, followed by those with higher risk classifications. After each step, allocation probability could be re‐evaluated, with further delisting if considered necessary to achieve a meaningful reduction in vPRA. However, granular classification of humoral risk per individual HLA‐specificity in highly sensitised patients is inherently complex. To address these complexities, working groups on sensitisation in transplantation, such as The European Working group on Guidelines for the Management of Graft Recipients (ENGAGE) and the working group on Sensitisation in Transplantation: Assessment of Risk (STAR), have stratified humoral risk based on CDC‐ or flow‐crossmatch results, Luminex single‐antigen bead (SAB) profiles, and sensitisation history [1, 2, 12]. While these frameworks are invaluable, they primarily infer the risk of a humoral response from the perspective of serological memory without directly assessing the cellular memory compartment.

HLA‐specific memory B‐cell (mBC) assessment could therefore enhance risk stratification, as mBC are key effectors of the memory response. Research showed that patients with preformed donor‐specific antibodies (DSA) and circulating mBC targeting the same HLA molecule had worse outcomes compared to patients with DSA alone [13, 14]. However, current methods for evaluating HLA‐specific mBC detect only circulating mBC, whereas these cells often reside in secondary lymphoid organs [15]. This limitation increases the risk of false‐negative results and reduces the clinical utility of these assays [16].

Studies in patients with systemic lupus erythematosus (SLE) showed that a 4‐to‐8 weeks course of belimumab, a BAFF‐inhibitor, mobilises mBC into circulation [17]. Flow‐cytometric analyses further detail two‐to fourfold increases in switched and unswitched memory B‐cell frequencies within 4 weeks of treatment, which contrasts decreases in all other circulating B‐cell and plasma cell subsets [18, 19]. Additionally, various previous studies in both SLE and in renal transplantation suggest that belimumab does not pose a significant safety risk compared to placebo [20, 21, 22, 23]. Thus, a short course of belimumab could potentially safely reduce the risk of false‐negative mBC detection and improve risk stratification. At our center, seven highly sensitised (vPRA > 85%) patients with poor (< 2%) allocation probability, who required delisting to achieve a reasonable chance of a donor match, were treated with a short course of off‐label belimumab after obtaining informed consent. The objective was to enhance allocation probability while minimising immunological risk by delisting only HLA‐specificities without detectable mobilised mBC, thereby reducing the risk of preformed DSA with associated cellular memory [13, 14]. In this study, we report on the effects of belimumab on detectable HLA‐specific mBC profiles.

## 2 Methods & Materials

### 2.1 Patient Treatment

Between 2022 and 2023, seven highly‐sensitised patients with limited allocation probability (< 2.0% matching donor frequency) received a four‐week course of 200 mg subcutaneous off‐label belimumab (Benlysta, GlaxoSmithKline LTD, Ireland) after providing informed consent. Belimumab was administered once weekly via autoinjector by patients or their caregivers under guidance from a physician. Six patients provided written informed consent for description in this case series. One patient died recently, 15 months after treatment discontinuation, and could not consent to the use of their medical data. Considering this report contains no uniquely identifying information, written consent for publication is not legally required posthumously [24, 25]. Reporting of these results was also approved under local IRB protocol 132597.

### 2.2 Calculation of vPRA and Allocation Probability

Both vPRA and allograft allocation probability were calculated using online Eurotransplant Reference Laboratory applications [26]. Allocation probability was estimated using the AB0 ET compatible calculator, which determines the frequency of matching donors among a population of 10,000 individuals typed at high resolution. This calculator accounts for the Acceptable Mismatch program rules on blood‐type compatibility, allowing patients with blood‐type B to receive offers from blood‐type 0, and those with blood‐type AB to receive offers from blood‐type A.

### 2.3 HLA Typing of Patients

Patient DNA was genotyped at high resolution (second field) for HLA‐A, B, C, DRB1, DRB3/4/5, DQA1, DQB1, DPA1, and DPB1 through next generation sequencing techniques (Illumina NGS, GenDx, Utrecht, the Netherlands).

### 2.4 In Vitro B‐Cell Culture and Supernatant Processing

The HLA‐specificity profile of circulating memory B‐cells was evaluated on the first day of treatment before the first belimumab injection and 4 weeks thereafter, using methods described by Karahan et al. [27] Briefly, peripheral blood mononuclear cells were isolated and polyclonally activated with 2.5 μg/mL Toll–like receptor 7/8 agonist (resiquimod [R848]; Sigma–Aldrich, St. Louis, MO) and 1000 IU/mL IL–2 (Proleukin, Novartis, the Netherlands). IgG from the supernatant was isolated, eluted, and concentrated after 10 days. Pre‐ and post‐treatment samples were processed simultaneously to reduce batch effects.

### 2.5 Assessment of HLA‐Specific Antibodies

Circulating HLA‐specific antibodies were assessed every 3 months per Eurotransplant regulations and on days that B‐cell memory assays were conducted. Class I and II HLA‐antibodies were detected using Lifecodes SAB kits (LSA; Werfen, Barcelona, Spain). Serum samples were treated with EDTA before HLA‐antibody testing. The following methodology applies to antibody detection in both regular serum and in IgG eluates resulting from stimulated memory B‐cells in our memory B‐cell assay. Data analysis was performed using MATCHIT! Software (Werfen, Barcelona, Spain), which calculates background‐corrected mean MFI (BCM), BCM divided by the raw MFI of the lowest ranked bead for a locus (BCR), and antigen‐density corrected BCR values (AD‐BCR). Bead positivity was defined per manufacturer's instructions: for class I, BCM > 1500, BCR > 3, AD‐BCR > 4 (minimum 2/3 parameters); for class II, BCM > 1500, BCR > 4, AD‐BCR > 5 (minimum 2/3 parameters). The mean MFI for a serological antigen is determined by averaging all corresponding allele‐specific beads, with at least one bead meeting the positivity criteria.

### 2.6 Statistics

Statistical analyses were conducted using R software (v4.2.0, R Foundation for Statistical Computing, Vienna, Austria). Categorical variables are presented as counts and percentages, while continuous variables are reported as medians with interquartile ranges (IQR).

HLA‐specificities were stratified using risk‐tier frameworks established by STAR and ENGAGE which categorise risk based on CDC positivity, flow‐crossmatch positivity, SAB profiles, and (potential) historical sensitisation [1, 2, 12]. To emulate these tiers for individual specificities, we applied mean fluorescence intensity (MFI) thresholds on the Lifecodes platform: > 12,000 MFI for virtual CDC positivity and > 7000 MFI for virtual flow crossmatch positivity. These thresholds align with empirical lab experience and published data supporting their predictive value for positive crossmatches [28, 29, 30].

Generalised estimating equations (GEE) were used to compare the proportion of HLA‐specificities eliciting a positive response in mBC profiling before and after treatment, incorporating patient identity as a covariate to account for multiple specificities per patient. A Gaussian family GEE model was applied to assess changes in MFI of positive antigens over time. This paired analysis aimed to indirectly determine whether circulating HLA‐specific mBC increased post‐treatment. To prevent qualitative increases in detectability from skewing the results, only HLA‐specificities with detectable mBC before treatment were included in the quantitative analysis. Lastly, we examined the effect of belimumab on circulating HLA‐antibody MFI to assess whether the drug influences resident plasma cells or activates rather than mobilises mBC in vivo. For clarity and consistency, MFI values from the mBC assay are referred to as “mBC‐MFI”, while those from serum Luminex assays are designated “serum MFI”.

## 3 Results

### 3.1 Patient Demographics

A collective summary of the seven cases is provided in Table 1. The median age was 55 years (IQR 50–57), and four patients were female. The median number of prior transplants was 2 (IQR 0–2) with 2 documented prior rejection episodes (IQR 1–2). The median number of pregnancies among females was 2 (IQR 0–3). Three patients had a history of blood transfusions. The median vPRA was 99.77 (IQR 99.30–99.90), with a median of 42 UA (IQR 27–92). Baseline median allocation probability was 0.07% (IQR 0.00–0.41). Data from SAB assays conducted prior to treatment were available, with testing performed up to a median of 2.8 years (IQR 1.5–15.9) before treatment initiation.

**TABLE 1:** Descriptive characteristics of the population.

	Total (frequency) or median [IQR]
Age (years)	55 [50–57]
Gender	
Male	3 (43%)
Female	4 (57%)
Primary renal disease	
Polycystic disease	2 (29%)
Glomerulonephritis	2 (29%)
Reflux nephropathy	1 (14%)
Crystal nephropathy	1 (14%)
Unknown	1 (14%)
Number of previous transplants	2 [0–2]
Number of documented previous rejections of previous transplants	2 [1–2]
Number of documented previous pregnancies in female patients	2 [0–3]
Known history of blood product transfusions	3 (43%)
Time since last known sensitising event	
(Years)	15.6 [3.2–19.2]
Unknown	1 (14%)
Eligible for AM at time of treatment	
Yes	4 (57%)
No	3 (43%)
Number of unacceptable antigens	42 [27–92]
vPRA (%)	99.77 [99.30–99.90]
Allocation probability (%)	0.07 [0.00–0.41]

*Note:* Allocation probability was calculated through the AB0 ET‐compatible calculator^26^.

Abbreviations: AM: acceptable mismatch program; ET: eurotransplant; IQR: interquartile range; vPRA: virtual panel‐reactive antibodies.

### 3.2 Qualitative Effect of Belimumab on Circulating HLA‐Specific Memory B‐Cells

Patients received belimumab and HLA‐specific mBC‐profiles were assessed before and after treatment. Following belimumab treatment, significantly broader HLA‐specific mBC‐profiles were observed (Table 2). Among specificities with serum MFI > 12,000, positivity rates after memory B‐cell culturing increased from 86% pre‐treatment to 99% post‐treatment (*p* = 0.0062). For specificities with serum MFI between 7000 and 11,999 and 3000–6999, positivity rates improved from 50% to 75% (*p* < 0.0001) and 51% to 81% (*p* < 0.0001) after memory B‐cell culturing, respectively. 99% of mBC‐specificities with serum MFI > 3,000 that were detected before treatment remained detectable after treatment. Twelve additional mBC‐specificities were identified post‐treatment among specificities with serum MFI < 3000, while nine became negative. This difference was not statistically significant (*p* = 0.51). Taken together, 136 of 176 (77%) HLA‐specificities with currently detectable circulating HLA‐antibodies with MFI < 12.000 demonstrated memory B‐cell reactivity, either before or after belimumab treatment. There was no significant increase in mBC detectability among HLA‐specificities with only historical antibodies or repeated HLA‐mismatches without prior antibodies (*p* = 0.14), or immunologically naïve specificities without any antibody history (*p* = 0.12).

**TABLE 2:** HLA‐specific memory B‐cell profiling assay results, stratified by serum antibody MFI for HLA‐A, B, C, DRB1, DRB3/4/5, DQB1, and DPB1 loci.

Antigen sensitisation tiers	Total antigens	Pre‐belimumab mBC positive	Post‐belimumab mBC positive	Concordance	*p* value
SAB MFI > 12,000	95	82/95 (86%)	94/95 (99%)	81/82 (99%)	0.0062
SAB MFI 7000—11,999	52	26/52 (50%)	39/52 (75%)	26/26 (100%)	< 0.0001
SAB MFI 3000—6999	69	35/69 (51%)	56/69 (81%)	35/35 (100%)	< 0.0001
SAB MFI 1000—2999	55	29/55 (53%)	32/55 (58%)	20/29 (69%)	0.51
Historic SAB positiveORRepeated HLA‐mismatchANDCurrent SAB negative	64	4/64 (6%)	6/64 (9%)	4/4 (100%)	0.14
SAB never positiveANDNo repeated HLA‐mismatch	435	26/435 (6%)	35/435 (8%)	17/26 (65%)	0.12

*Note:* Sensitisation strength criteria by circulating antibody MFI are based on the highest Luminex SAB MFI result measured within the last 6 months up to treatment initiation on the Lifecodes platform. Concordance implies positive agreement between both mBC‐profiling moments for HLA‐specificities that elicited positivity in the pre‐belimumab assay. P‐values are calculated through generalised estimated equations (GEE).

Abbreviations: CDC: complement dependent cytotoxicity assay; mBC: memory B‐cell; MFI: mean fluorescence intensity; SAB: single antigen bead assay.

### 3.3 Quantitative Effect of Belimumab on Circulating HLA‐Specific Memory B‐Cells

**FIGURE 1:**
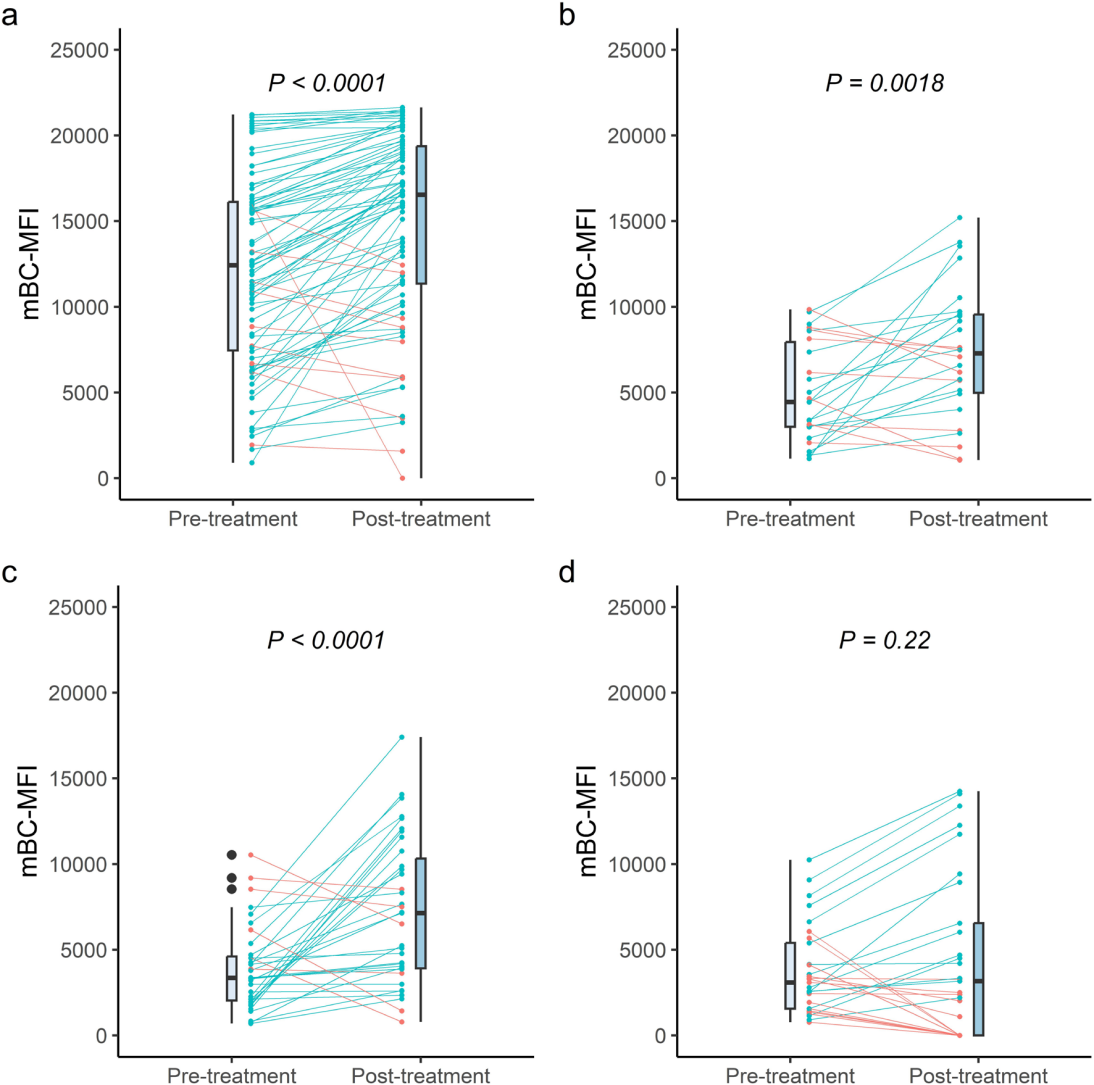
The mean fluorescence intensity of HLA‐antibodies derived from memory B‐cells for unacceptable HLA‐specificities, stratified by circulating antibody MFI. (A) Antigens with> 12,000 circulating antibody MFI (B) Antigens with circulating antibody MFI between 7000—11,999. (C) Antigens with circulating antibody MFI between 3000—6999. (D) Antigens with circulating antibody MFI between 1000—2999. Only specificities that were positive in the memory B‐cell assay before treatment are included in this analysis. Blue lines indicate an increase in mBC‐MFI after treatment, compared to pre‐belimumab, red lines indicate a decrease in mBC‐MFI compared to pre‐belimumab. *p*‐values are calculated through generalised estimated equations. mBC‐MFI: Memory B‐cell assay mean fluorescence intensity.

**FIGURE 2:**
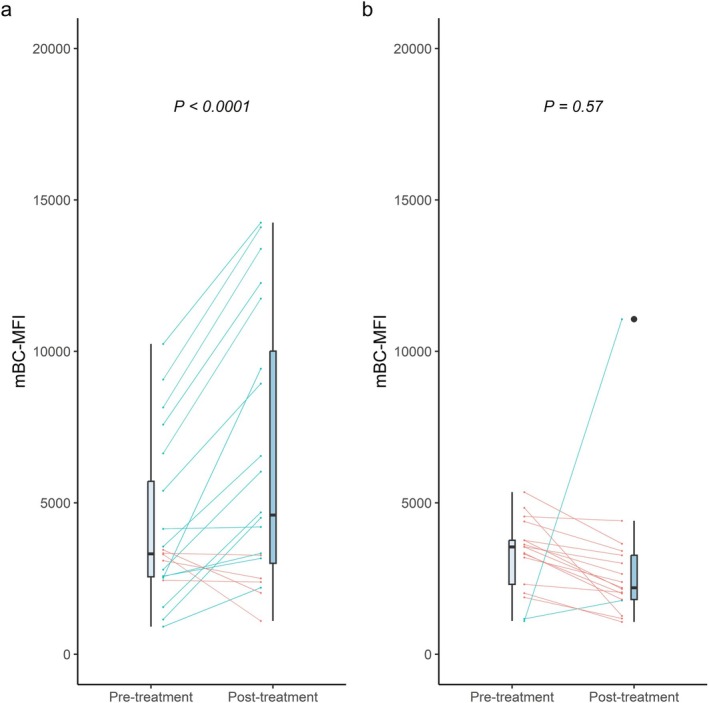
The mean fluorescence intensity of HLA‐antibodies derived from memory B‐cells for HLA specificities that were positive in both pre‐treatment and post‐treatment mBC profiling. (A) Antigens with circulating antibody MFI between 1000—2999. (B) Antigens with no history of detectable HLA‐antibodies. Blue lines indicate an increase in mBC‐MFI after treatment, compared to pre‐treatment, red lines indicate a decrease in mBC‐MFI compared to pre‐treatment. The *p*‐values are calculated through generalised estimated equations. MFI: Mean fluorescence intensity.

Next, we assessed the effect of belimumab on MFI values of HLA‐antibodies produced by mBC in our mBC‐assay. As shown in Figure 1, a significant increase in mBC‐MFI was observed. Specificities with serum MFI > 12,000 demonstrated a marked rise in mBC‐MFI (median [IQR]; pre‐treatment: 12,429 [7,452–16,113] versus. post‐treatment: 16,548 [11,355–19,372], *p* < 0.0001). Similarly, HLA‐specificities with serum MFI between 7000 and 11,999 showed a significant increase (pre‐treatment: 4445 [3,001–7,946] versus. post‐treatment: 7288 [4,978–9,548], *p* = 0.0018), as did specificities with serum MFI between 3000 and 6999 (pre‐treatment: 3362 [2,039–4,612] versus. post‐treatment: 7147 [3,910–10,317], *p* < 0.0001). No significant change in mBC‐MFI was observed for specificities with serum MFI < 3000 as some positive mBC‐specificities became undetectable post‐belimumab (*p* = 0.22). However, mBC‐MFI did increase significantly when the analysis was restricted to specificities detectable both before and after treatment (pre‐treatment: 3317 [2,556—5,710] versus. post‐treatment: 4596 [3,002—10,009], *p* < 0.0001). (Figure 2A) A similar analysis for HLA‐specificities without (history of) detectable HLA‐antibodies showed no significant difference (*p* = 0.57). (Figure 2B).

### 3.4 Effect of Belimumab on Circulating HLA‐Antibodies

**FIGURE 3:**
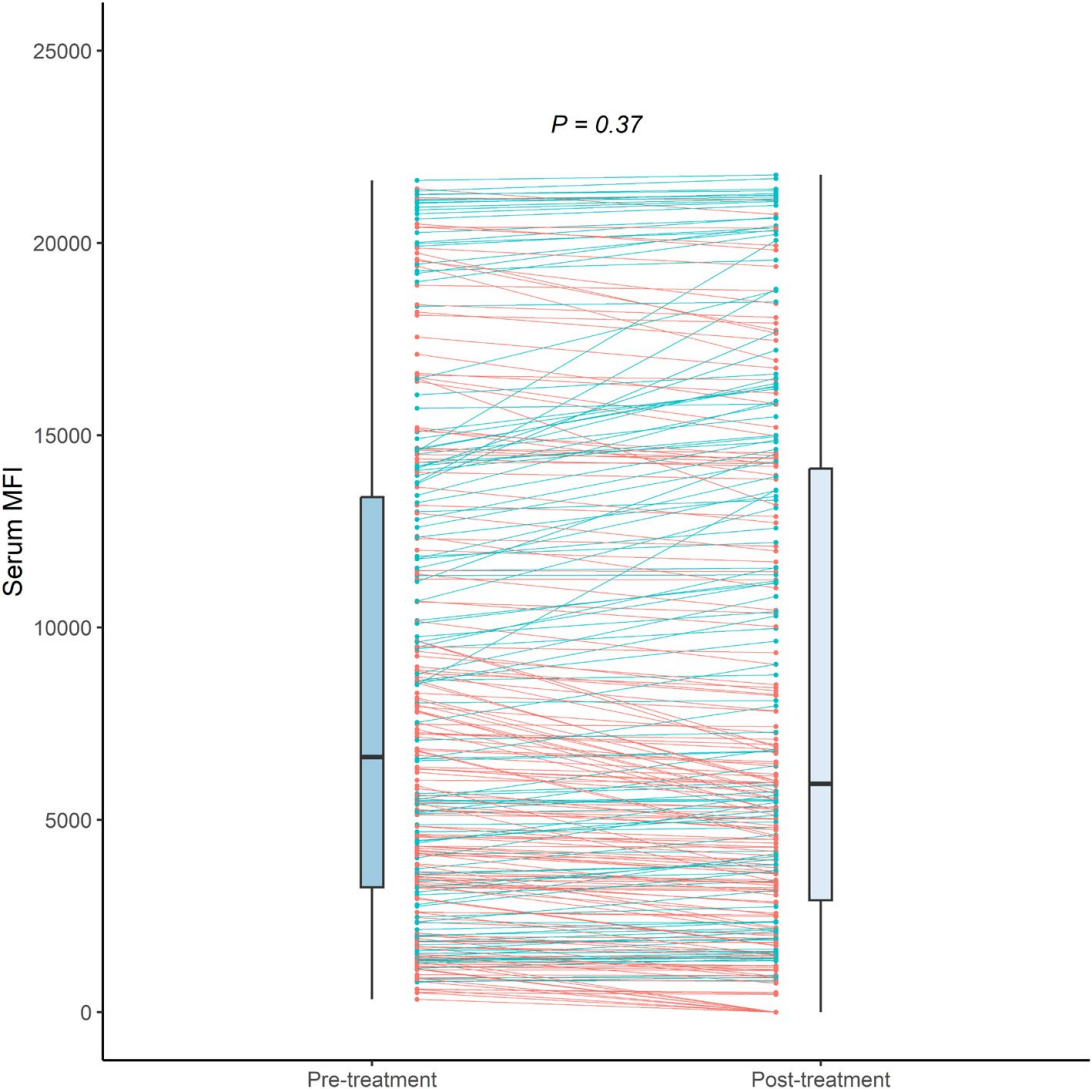
The mean fluorescence intensity (MFI) of circulating HLA‐antibodies in serum, before and after treatment with belimumab. Blue lines indicate an increase in MFI after treatment, compared to pre‐treatment; red lines indicate a decrease in MFI compared to pre‐treatment. The *p*‐value is calculated through generalised estimated equations. Serum MFI: Serum antibody mean fluorescence intensity.

There was no significant effect of belimumab on circulating serum HLA‐antibody MFI, contrasting the observed mBC‐MFI increase (pre‐treatment: 6633 [3,244—13,389] versus. post‐treatment: 5937 [2,910—14,134], *p* = 0.37). (Figure 3).

### 3.5 Potential Effects of Utilising Memory B‐Cell Profiling in Delisting and Transplant Outcomes

To assess how using our proposed strategy in delisting could clinically affect the chance of a donor offer, we analysed how delisting unacceptable antigens without detectable memory B‐cell reactivity improves vPRA and allocation probability (Supplementary Results; Table S1).

In addition, the supplementary results (page 3) detail excellent transplant outcomes of three of our cases who were transplanted since treatment.

### 3.6 Adverse Events and Other Possible Sensitising or mBC Mobilising Events

This short course of belimumab was well‐tolerated with one possibly related adverse event reported within 6 months after treatment (non‐severe SARS‐CoV2 infection). During treatment, no patients received blood product transfusions, became pregnant, underwent transplantation, or received vaccinations.

## 4 Discussion

This report demonstrates that four weekly injections of belimumab may enhance the detection of HLA‐specific mBC with concurrent circulating HLA‐antibodies. The broader profiles detected after treatment further underscore previous reports warning that HLA‐specific mBC may otherwise be missed, as these cells are predominantly located in secondary lymphoid organs [15, 16].

Moreover, the increased mBC‐MFI for specificities with detectable antibodies indirectly suggests that the number of circulating HLA‐specific memory B cells increased. These findings align with multiple prior large‐scale studies in SLE patients, which established that belimumab induces a rapid increase in circulating memory B cells. Pooled analyses from multiple placebo‐controlled trials involving thousands of patients demonstrated that memory B‐cell levels approximately double within the first 4 weeks of treatment [17]. Single‐cell RNA sequencing analyses from these studies support a redistribution of memory B cells from lymphoid and inflamed tissues into the peripheral blood, rather than increased activation or proliferation [18]. Specifically, treated cells exhibited downregulation of genes associated with immune activation, antigen processing, and migration. Moreover, cell cycle analyses indicated a reduction in proliferating memory B cells, and proliferation‐associated transcripts were not expressed. Taken together, these transcriptomic signatures are consistent with a less activated, non‐proliferating phenotype and suggest impaired lymphoid homing as the most plausible mechanism of action [18].

Our own observations in this study align with the data in our auto‐immunity studies. First, no increase in serum HLA antibody MFI was observed post‐treatment, suggesting that belimumab did not activate pre‐existing memory B‐cell clones or resident plasma cells in vivo. Second, no significant change in positive responses was observed in our mBC assay for HLA specificities without known prior sensitisation, arguing against non‐specific assay interference or in vitro activation effects. Third, no known sensitising or mobilising events (e.g., vaccinations or blood transfusions) occurred during the brief evaluation window.

Taken together, we feel that our results are consistent with belimumab‐induced mobilisation of HLA‐specific memory B‐cells into peripheral blood, leading to enhanced detection of previously undetected specificities. These findings illustrate the potential of leveraging this physiological effect to improve identification of memory B‐cell responses in sensitised individuals.

We further observed that belimumab did not lead to enhanced mBC detection for all UA, not even for some UA with serum antibody MFI up to 12,000. Mechanistically, this may reflect discrepancies in the prevalence of HLA‐antibody producing plasma cells and mBC targeting the same epitope. Although both cell types derive from a common naïve B‐cell precursor, their differentiation does not occur in a 1:1 ratio [31]. Experimental research further showed that plasma cells and mBC can survive independently [32]. This may explain the presence of circulating HLA‐antibodies without corresponding mBC, and vice versa, as observed by us and others [13, 14].

It is also possible that belimumab did not mobilise all HLA‐specific mBC into circulation within the four‐week time frame, or that sampling error due to limited venous sampling still leaves a potential for false‐negative detection. Assessing this directly is challenging without an available gold standard for comparison. Nonetheless, the detection of nearly 100% of mBC corresponding to virtual CDC‐reactive specificities post‐treatment suggests enhanced detection performance for specificities where the presence of B‐cell memory seems likely. In contrast, no significant increase in mBC detection occurred for specificities without prior history of HLA‐specific antibodies or known exposure, indicating that belimumab does not enhance detection rates for all HLA‐specificities indiscriminately.

Interestingly, we detected HLA‐specific mBC reactivity directed against a limited number of antigens without a documented history of sensitisation through pregnancy or transplantation and without a history of HLA‐specific antibodies. These findings could reflect false‐positive results, potentially due to the presence of denatured antigens in bead‐based assays, which is a known limitation [33]. However, 88% (23/26) of such specificities pre‐treatment and 89% (31/35) post‐treatment were found in a single individual (Patient 5), suggesting a patient‐specific phenomenon. Further analysis using HLAMatchmaker [34] revealed that these antigens shared four eplet targets (71TTS, 76ESN, 80I, 80 K) with one HLA‐C and two HLA‐B mismatches, to which the patient was exposed through previous pregnancies over two decades prior, as confirmed by HLA typing of the patient's children and their father. This supports the hypothesis that these mBC may reflect cross‐reactive memory responses from prior sensitisation events that are not captured by conventional SAB assays. These findings may highlight the added value of mBC analysis in uncovering potentially clinically relevant reactivities that could be missed by serum‐based antibody testing alone.

These observations also underscore a broader limitation in relying solely on documented immunisation history or circulating antibody profiles for risk assessment in highly sensitised patients. In individuals with extensive sensitisation and numerous unacceptable antigens, it is often difficult to trace specific reactivities to defined exposures due to undocumented events or epitope‐level cross‐reactivity. Direct memory B‐cell profiling may therefore offer complementary insight.

Our study thus suggests that belimumab may enhance the diagnostic validity of HLA‐specific mBC analysis, yet its clinical utility remains unknown. A potential application lies in delisting HLA‐specificities with detectable antibodies but no detectable mBC. Notably, approximately 20%–25% of specificities with circulating antibody MFI < 12,000 remained without detectable mBC following belimumab treatment. Therefore, we envision this approach to refine risk stratification in delisting strategies for highly sensitised patients. Two recent Spanish studies reported on improved transplant rates following delisting based on circulating HLA‐antibody MFI [9, 35]. However, AMR remained a major complication. Garcia‐Jimenez et al. [9] reported AMR rates of 30%–40% in patients with DSA> 5000 MFI, while Comins‐Boo et al. found AMR in all five patients with available post‐transplant biopsies after transplantation with DSA < 5000 MFI [35]. Two additional studies similarly reported AMR or early graft loss rates of 20%–30% in cases of low DSA MFI [36, 37]. If donor‐specific mBC contributes to these risks, our strategy of mBC mobilisation may aid in risk stratification by limiting the risk of false‐negative mBC analysis.

While memory B‐cell profiling may enable more precise delisting, it may also restrict delisting options, as certain HLA‐specificities with detectable mBC responses may no longer qualify despite the absence of circulating antibodies. Furthermore, the clinical significance of memory B‐cell reactivity in the absence of concurrent serum HLA antibodies remains uncertain, highlighting the need for prospective studies to guide interpretation and implementation.

We envision that a stepwise delisting approach that accounts for both HLA‐antibodies and HLA‐specific mBC could optimise the balance between humoral risks, allocation probability, and the competing risk of dialysis‐associated mortality. First, even small improvements in allocation probability by delisting “low risk” specificities without detectable memory can significantly impact waiting times. US registry data show that lowering vPRA from > 99.9% to < 99.9% nearly doubles the likelihood of deceased donor transplantation within 3 years (26% versus 47%) [5]. Similar trends were observed in Korean and Australian data for patients with vPRA above or below 99% [6, 7]. Eurotransplant data further support this, demonstrating that patients with a 0.5% matching donor frequency have an 80% chance of transplantation within 3 years, compared to 60% for those with a frequency of 0.1%–0.5% and less than 30% for those below 0.1% [4].

Second, if delisting “low‐risk” specificities without detectable mBC does not sufficiently lower vPRA or improve allocation probability, this approach could be combined with desensitisation strategies (e.g., imlifidase) for higher‐risk specificities. Our data suggest this is feasible, as 25% of virtually crossmatch‐positive HLA‐specificities with antibody MFI between 7000 and 12,000 exhibited no reactivity in the mBC assay after belimumab treatment.

The potential improvements in vPRA and allocation probability we outline in the supplementary results further underscore the feasibility of this stepwise approach.

Lastly, for patients with broad HLA‐specific memory B‐cell repertoires, a desensitisation strategy could be envisioned where these cells are initially mobilised with belimumab and then depleted through rituximab, likewise to treatment modalities in auto‐immunity [38, 39, 40]. Future studies in highly sensitised waitlisted patients, combining such strategies with emerging therapies, may further clarify its clinical utility [41]. To that end, an investigator‐initiated study, BE‐MOBILYZED (CTIS identifier 2023–508116–32‐00), is currently being designed.

Our study has several limitations. First, this was not a clinical trial, but an evaluation of a case series involving seven highly‐sensitised patients with poor transplant prospects. These patients received off‐label treatment with informed consent, as permitted under Dutch law. This context limited follow‐up analyses for research purposes, such as tracking mBC fate after treatment cessation. Second, this study focused solely on humoral memory responses. The presence of donor‐specific memory T‐cells was not assessed; however, our strategy does not preclude complementary assessment of T‐cell memory. Third, we utilised relatively arbitrary cut‐offs in SAB MFI to determine virtual crossmatch positivity for individual UA. However, performing physical crossmatches to accurately determine which HLA specificities will cause reactivity is infeasible. These MFI cut‐offs were not intended to stratify humoral risk but rather to emulate the risk tiers proposed by STAR and ENGAGE. Any cut‐off, whether based on MFI or antibody titre, for this purpose will be arbitrary, and we therefore also did not perform titrated assays. Lastly, the number of patients precluded meaningful analysis of long‐term transplant outcomes. A larger population is required to adequately assess the long‐term clinical impact of our proposed strategy, and further trials are thus needed.

In conclusion, our case‐series demonstrates that belimumab reveals a broader profile of HLA‐specific memory B‐cells. This strategy may pave the way for a new paradigm in pretransplant immunological risk‐stratification, as it enables a more refined assessment of HLA‐specific memory B‐cell profiles, potentially reducing the risk of memory responses in highly‐sensitised renal transplant recipients. The clinical implications of this strategy for transplant outcomes, as well as the necessity and extent of tailored desensitisation protocols in such cases require further investigation.

## Conflicts of Interest

Y.K. Onno Teng has previously received grant support from GlaxoSmithKline and Aurinia Pharmaceuticals and consulting fees from Kezar Life Sciences, GlaxoSmithKline, Aurinia Pharmaceuticals, and Novartis. Aiko P.J. de Vries received consulting and speaker fees from Novartis, Neovii, Hansa, Sandoz, Takeda, Sanofi, Astellas, all of which went to his employer and none to personal bank accounts. All remaining authors declare no conflicts of interest.

## Supporting information


Data S1.

## Data Availability

Due to the sensitive nature of (genetic) HLA data upon which the results of this study are based, data cannot be publicly shared. Certain types of data may be shared for research purposes upon request to the corresponding author.
