# Supplementary material for: Belimumab to Aid Pre‐Transplant Immunological Risk‐Stratification by Uncovering Broader HLA‐Specific Memory B‐Cell Profiles
Source: HLA. 2025 Jun 16;105(6):e70285. doi: 10.1111/tan.70285 (PMC12169098; doi:10.1111/tan.70285)
Supplement: Supplementary file 1 — Data S1. [file TAN-105-e70285-s001.docx]

**Supplementary Materials**

**Table of Contents**

**Supplementary methods2**

**Stepwise delisting of unacceptable antigens 2**

**Supplementary Results3**

**Effects of stepwise delisting on vPRA and allocation probability 3**

**Transplant outcomes and organ offers3**

**Supplemental Table 14**

**Supplementary References5**

**Supplementary Methods and Materials
Stepwise delisting of unacceptable antigens**At our center, all HLA-specificities with detectable serum antibodies, whether historical or current, are initially listed as unacceptable for kidney transplant candidates. To assess the potential utility of incorporating memory B-cell profiling into the delisting process, we performed a stepwise delisting analysis using a standardized framework informed by risk-tier stratification guidelines by STAR and ENGAGE.^1-3^. To emulate these tiers for individual unacceptable HLA-specificities, we used SAB mean fluorescence intensity (MFI) thresholds on the Lifecodes platform: >12,000 MFI for virtual CDC-positivity and >7,000 MFI for virtual flow-crossmatch positivity. These MFI cut-offs reflect empirical lab experience and research supporting their predictive value for positive crossmatches with this vendor.^4-6^
To extend this framework, we incorporated data from the HLA-specific mBC assay. Only specificities without detectable mBC reactivity, either before or after belimumab treatment, were considered for potential delisting. We then simulated a stepwise delisting protocol, beginning with the lowest immunologic risk, based on our local institutional protocol:

**Tier 1 (Local protocol)**: Specificities without detectable mBC, with current SAB MFI <3,000, excluding repeat HLA-mismatches from prior transplants or pregnancies.

**Tier 2**: Additional specificities without detectable mBC and current SAB MFI between 3,000–6,999.

**Tier 3:** Additional specificities without detectable mBC and current SAB MFI between 7,000–11,999.

At each step, we calculated the reduction in virtual panel reactive antibody (vPRA) and the corresponding increase in allocation probability within the Eurotransplant region. This analysis aimed to evaluate the theoretical benefit of integrating mBC profiling into current listing practices.

**Supplementary Results**

**Effects of stepwise delisting on vPRA and allocation probability**Six patients had unacceptable antigens that could be delisted under standard clinical protocol, taking account for detectable mobilized memory. This would reduce vPRA levels in four patients. (Supplemental Table 1) For patients one, three, five, and six, vPRA would decrease by 21.03%, 0.41%, 1.40%, and 0.21%, respectively, improving allocation probability by 10.31%, 0.18%, 0.46%, and 0.13% respectively. Further delisting beyond our local protocol by delisting all antigens with serum antibody MFI <7,000 without detectable mBC could further reduce vPRA by 78.87%, 1.50%, and 19.0% for patients one, three, and five, respectively. This would increase allocation probability by 37.54%, 0.91%, and 7.46% respectively. In patient five, delisting all antigens with serum antibody MFI up to 12,000 without detectable mBC would further lower vPRA to 7.89% and boost allocation probability to 36.95%.

**Transplant outcomes and organ offers**One patient received two donor offers within four months of active waiting time after delisting, but both were declined due to poor quality. A third offer, expressing two antigens (HLA-A*32:01 and HLA-DPA1*01:03/DPB1*02:01) with historical antibodies but no detectable memory, was accepted at 21 months post-treatment. The patient experienced immediate graft function with no signs of rejection or DSA rebound to date at nine months post-transplant. Two other patients were transplanted with grafts that expressed no delisted antigens at twelve- and 18-months post-treatment. Donor-specific memory was negative for both, and no HLA-DSA formation has been observed at currently eight- and twelve-months post-transplant with stable graft function.

| **Unacceptable Antigens** | | | | | | | |
| --- | --- | --- | --- | --- | --- | --- | --- |
| **Delisting criteria** | Patient 1 | Patient 2 | Patient 3 | Patient 4 | Patient 5 | Patient 6 | Patient 7 |
| **Baseline** | 42 | 96 | 27 | 25 | 27 | 57 | 92 |
| **Current antibody MFI <3000; No repeated mismatches** | 5 | 85 | 25 | 25 | 22 | 52 | 78 |
| **Current antibody MFI < 7,000** | 0 | 81 | 21 | 25 | 15 | 52 | 78 |
| **Current antibody MFI < 12.000** | 0 | 80 | 21 | 25 | 3 | 52 | 78 |
| **vPRA** | | | | | | | |
| **Delisting criteria** | Patient 1 | Patient 2 | Patient 3 | Patient 4 | Patient 5 | Patient 6 | Patient 7 |
| **Baseline** | 99.90 | 100 | 99.3 | 99.27 | 95.6 | 99.77 | 100 |
| **Current antibody MFI <3000; No repeated mismatches** | 78.87 | 100 | 98.89 | 99.27 | 94.2 | 99.56 | 100 |
| **Current antibody MFI < 7,000** | 0 | 100 | 97.39 | 99.27 | 75.2 | 99.56 | 100 |
| **Current antibody MFI < 12.000** | 0 | 100 | 97.39 | 99.27 | 7.89 | 99.56 | 100 |
| **Allocation Probability** | | | | | | | |
| **Delisting criteria** | Patient 1 | Patient 2 | Patient 3 | Patient 4 | Patient 5 | Patient 6 | Patient 7 |
| **Baseline** | 0.06 | 0.00 | 0.41 | 0.3 | 1.69 | 0.07 | 0.00 |
| **Current antibody MFI <3000; No repeated mismatches** | 10.37 | 0.00 | 0.59 | 0.3 | 2.15 | 0.20 | 0.00 |
| **Current antibody MFI < 7,000** | 47.91 | 0.00 | 1.50 | 0.3 | 9.51 | 0.20 | 0.00 |
| **Current antibody MFI < 12.000** | 47.91 | 0.00 | 1.50 | 0.3 | 36.95 | 0.20 | 0.00 |

*Supplemental Table 1: Effect of delisting on unacceptable antigens, vPRA, and allocation probability*
The impact of delisting on the number of unacceptable antigens, vPRA and allocation probability. All HLA-specificities with either historical or current single antigen bead reactivity on serum are defined as unacceptable at baseline. Only HLA-specificities without detectable memory, as defined by our memory B-cell assay, are eligible for delisting in this analysis. Allocation probability was calculated through the AB0 ET-compatible calculator.*^7^* ET: Eurotransplant; MFI: Mean fluorescence intensity.

**Supplementary references**

1. Bestard O, Couzi L, Crespo M, Kessaris N, Thaunat O. Stratifying the humoral risk of candidates to a solid organ transplantation: a proposal of the ENGAGE working group. Transpl Int. 2021;34(6):1005-18.

2. Bestard O, Thaunat O, Bellini MI, Bohmig GA, Budde K, Claas F, et al. Alloimmune Risk Stratification for Kidney Transplant Rejection. Transpl Int. 2022;35:10138.

3. Tambur AR, Campbell P, Claas FH, Feng S, Gebel HM, Jackson AM, et al. Sensitization in Transplantation: Assessment of Risk (STAR) 2017 Working Group Meeting Report. Am J Transplant. 2018;18(7):1604-14.

4. Baranwal AK, Bhat DK, Goswami S, Agarwal SK, Kaur G, Kaur J, et al. Comparative analysis of Luminex-based donor-specific antibody mean fluorescence intensity values with complement-dependent cytotoxicity & flow crossmatch results in live donor renal transplantation. Indian J Med Res. 2017;145(2):222-8.

5. Flynn PA, Fernando S, Worthington JE, Poulton KV. Predicting flow cytometry crossmatch results from single-antigen bead testing. Int J Immunogenet. 2024;51(2):93-9.

6. Locke AF, Hickey M, Valenzuela NM, Butler C, Sosa R, Zheng Y, et al. Virtual and Reality: An Analysis of the UCLA Virtual Crossmatch Exchanges. Transplantation. 2023;107(8):1776-85.

7. Eurotransplant. Eurotransplant vPRA and donor frequency calculators. ETRL reference database v4. 2023 [Available from: [www.etrl.org](file:///\\vf-lumc-home.lumcnet.prod.intern\lumc-home$\dajvandenbroek\Imlifidase%20belimumab\Belimumab%20artikel\www.etrl.org)].
